# Circadian disruption reduces MUC4 expression via the clock molecule BMAL1 during dry eye development

**DOI:** 10.1038/s12276-024-01269-0

**Published:** 2024-07-02

**Authors:** Hao Zeng, Xue Yang, Kai Liao, Xin Zuo, Lihong Liang, Dalian He, Rong Ju, Bowen Wang, Jin Yuan

**Affiliations:** State Key Laboratory of Ophthalmology, Zhongshan Ophthalmic Center, Sun Yat-sen University, Guangdong Provincial Key Laboratory of Ophthalmology Visual Science, Guangzhou, 510060 China

**Keywords:** Experimental models of disease, Chronic inflammation, Circadian rhythms

## Abstract

Circadian disruption, as a result of shiftwork, jet lag, and other lifestyle factors, is a common public health problem associated with a wide range of diseases, such as metabolic disorders, neurodegenerative diseases, and cancer. In the present study, we established a chronic jet lag model using a time shift method every 3 days and assessed the effects of circadian disruption on ocular surface homeostasis. Our results indicated that jet lag increased corneal epithelial defects, cell apoptosis, and proinflammatory cytokine expression. However, the volume of tear secretion and the number of conjunctival goblet cells did not significantly change after 30 days of jet lag. Moreover, further analysis of the pathogenic mechanism using RNA sequencing revealed that jet lag caused corneal transmembrane mucin deficiency, specifically MUC4 deficiency. The crucial role of MUC4 in pathogenic progression was demonstrated by the protection of corneal epithelial cells and the inhibition of inflammatory activation following MUC4 replenishment. Unexpectedly, genetic ablation of BMAL1 in mice caused MUC4 deficiency and dry eye disease. The underlying mechanism was revealed in cultured human corneal epithelial cells in vitro, where BMAL1 silencing reduced MUC4 expression, and BMAL1 overexpression increased MUC4 expression. Furthermore, melatonin, a circadian rhythm restorer, had a therapeutic effect on jet lag-induced dry eye by restoring the expression of BMAL1, which upregulated MUC4. Thus, we generated a novel dry eye mouse model induced by circadian disruption, elucidated the underlying mechanism, and identified a potential clinical treatment.

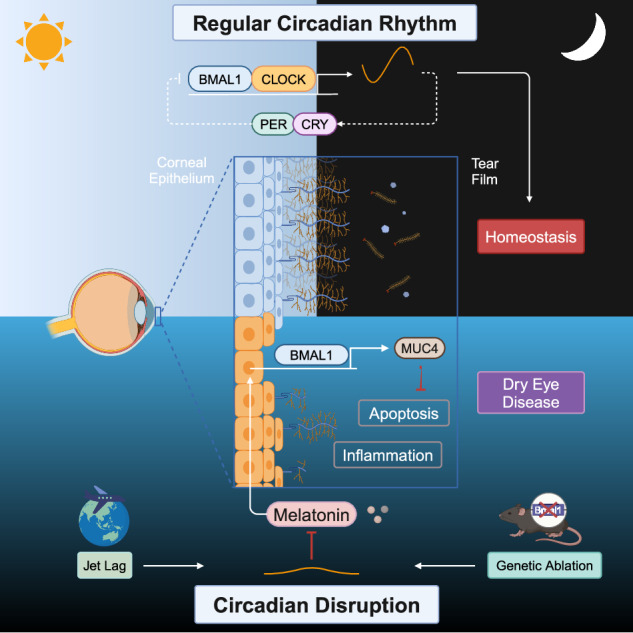

## Chronic jet lag induces dry eye: MUC4 deficiency revealed

Dry eye disease, a long-term issue causing discomfort and vision problems, impacts millions globally. In this research, scientists studied how disturbances in our internal clock contribute to DED. Researchers made the mice experience an 8-hour shift in their day-night cycle every 3 days, imitating chronic jet lag. The findings showed that chronic jet lag resulted in a significant decrease in MUC4 expression in the cornea, leading to DED symptoms. Supplementing with MUC4 or treating the mice with melatonin, eased these symptoms. This indicates that disruptions to our internal clock can directly affect eye health by impacting key protective proteins in the eye. Researchers conclude that maintaining a healthy internal clock is vital for eye health and that treatments targeting internal clock disruptions could help DED patients.

This summary was initially drafted using artificial intelligence, then revised and fact-checked by the author.

## Introduction

Dry eye disease (DED) is a common, multifactorial, and chronic ocular surface disease that affects the quality of life of patients, causes visual impairment, and results in a socioeconomic burden^1^. Symptoms and signs of DED can appear in isolation or in association with systemic diseases, such as Sjögren syndrome, diabetes, rosacea, and lifestyle-related disorders^2^. Lifestyle-related dry eye has become increasingly widespread in recent years, and the impact of lifestyle challenges on dry eye has attracted increased amounts of attention^3^. Several clinical studies have identified associations between DED and sleep disorders^4^, anxiety^5^, inactivity^6^, smoking^7^, and the use of visual display terminals^8^.

Many large population-based studies have shown that sleep disorders are associated with the symptoms, signs, and severity of DED^9–11^. A multihospital cross-sectional study showed that the use of sleeping tablets was associated with DED^12^. Sleep disorders include a wide range of heterogeneous conditions, one of the most common of which is insomnia^13^. To explore the underlying mechanisms of sleep deficiency in DED, a recent study used a “stick over water” approach to establish a sleep deprivation mouse model. These researchers found that sleep deprivation primarily caused damage to the function of the lacrimal gland, which resulted in decreased aqueous tear secretion and increased corneal epithelial defects^14^. Therefore, sleep deprivation is a successful model for inducing DED. Nonetheless, sleep deprivation does not adequately mimic real-world sleep disorders.

Another class of sleep disorders is circadian rhythm sleep-wake disorders^15^. As society evolves, populations affected by circadian-related factors such as shift work and jet lag are expanding annually^16^. The circadian rhythm is a cyclic variation in behavior and physiology that accommodates daily recurring environmental changes^17^. The circadian rhythm is controlled by an autonomous, intrinsic time-keeping system called the circadian clock, which is located in nearly every cell. This molecular clock consists of interlocking transcription–translation feedback loops^18^. The initiators that drive the circadian cycle are the transcription factor brain and muscle ARNT-like 1 (BMAL1; also known as ARNTL) and the circadian locomotor output cycle kaput (CLOCK)^19^. Deletion of BMAL1 in mice results in the absence of all molecular and behavioral circadian phenotypes^20^. In addition, when the circadian clock phase does not match the external time, environmental cues called zeitgebers determine the phase of the circadian clock. This process is referred to as entrainment. Light is the dominant zeitgeber that can reset or entrain endogenous rhythms^21^. In contemporary society, it is common for humans to be erroneously exposed to zeitgebers, which disrupt circadian homeostasis and cause metabolic disorders^22^, neurodegenerative diseases^23^, and cancer^24^. Furthermore, a recent study suggested that a shift in the light cycle phase modified the circadian rhythm of murine lacrimal glands^25^. However, whether circadian disruption causes DED and the underlying mechanism remain unknown.

Therefore, in the present study, we established a circadian disruption mouse model through a time shift to explore changes in the ocular surface. Furthermore, we investigated the exact mechanism of circadian disruption-induced DED in mice using BMAL1 knockout (KO) mice and in vitro using human corneal epithelial cells (HCECs).

## Materials and methods

### Establishment of animal models and intervention

All animal experiments adhered to the Association for Research in Vision and Ophthalmology Statement for the Use of Animals in Ophthalmic and Vision Research and were approved by the IACUC of Zhongshan Ophthalmic Center (Guangzhou, China; approval ID: 2020-138).

Eight-week-old female C57BL/6 mice were provided by Beijing Vital River Laboratory Animal Technology Co., Ltd. (Beijing, China). Before the establishment of the circadian disruption mouse model, the mice were kept under constant light‒dark cycles with the lights turned on at 8 a.m. (zeitgeber time 0, ZT0) and off at 8 p.m. (zeitgeber time 12, ZT12). To establish the jet lag model, mice were exposed to the condition which light was set for an advance of 8 h every 3 days as previously described^26^. For example, from Day 1 to Day 3, the light was turned on at 12 a.m. and turned off at 12 p.m. Then, for an advance of 8 h, the light was turned on at 4 p.m. on Day 3 and turned off at 4 a.m. on Day 4. The control group mice were subjected to constant light-dark conditions. In this study, two in vivo interventions were performed during the establishment of the chronic jet lag model. For supplementation with MUC4, 8-week-old female mice were subconjunctival injected with 5 μl of recombinant human MUC4 protein (rhMUC4, 20 ng/μl; Abnova, Taipei, China) or vehicle control (PBS, Servicebio, Wuhan, China) on Days 0, 8, 16, and 24 during the establishment of the chronic jet lag model. For circadian rhythm recovery treatment, melatonin (MT, 10 mg/kg, Sigma, St. Louis, USA) or vehicle control (PBS) was injected intraperitoneally into the mice at 9 a.m. every day during the induction of jet lag. Samples were collected when the mice with jet lag were in the same dark-light cycle as the control mice.

For analysis of the exact mechanism of circadian disruption in DED, 12-week-old BMAL1 KO mice were used in this study. Eight-week-old heterozygous BMAL1 (B6.129-Bmal1^tm1Bra^/J) mice on the C57BL/6 background were purchased from Jackson Laboratories (Bar Harbor, ME, USA), and we established a breeding colony of heterozygous BMAL1 mice. Wild-type (WT) mice and BMAL1 KO mice were bred in-house.

### Phenol red thread test

The volume of aqueous tear secretion was quantified with phenol red thread (Jingming, Tianjin, China). Briefly, a cotton thread containing phenol red dye was gently placed on the palpebral conjunctiva at approximately one-third of the distance from the lateral canthus of the eyelid for 15 s. Afterward, the lengths of the tear-wetted threads were recorded in millimeters. The test was performed at ZT8 on Day 30 to compare the tear secretion volume between the control and jet lag groups. The tests were performed at ZT0, ZT6, ZT12, ZT18, and ZT24 on Day 30 when comparing secretion rhythms.

### Corneal fluorescein staining

Corneal fluorescein staining was performed to assess the degree of corneal epithelial defects in the mice. Briefly, 0.25% fluorescein sodium (Jingming, Tianjin, China) was administered to the conjunctival sac, and the eyes were rinsed with normal saline after several blinks. The corneal images were taken using a cobalt blue filter under a slit-lamp microscope image system (SL-D7/DC-3/MAGENet; Topcon, Tokyo, Japan). The percentage of corneal defect area was determined by ImageJ software (version 1.53a; National Institutes of Health, Bethesda, MD, USA). The corneal defect coverage area (%) = (fluorescein sodium-positive area/whole cornea) * 100%.

### Corneal lissamine green staining

Lissamine green staining was used to detect mucin deficiency and ocular surface damage. After the fluorescein sodium was removed by washing with normal saline, 1% lissamine green (Sigma) was added to the conjunctival sac. After the animals had blinked several times, the eyes were rinsed with normal saline. The corneal images were taken using a cobalt blue filter under a slit-lamp microscope image system. Lissamine green staining in the cornea was scored using a scoring system ranging from 0 to 9^27^. The corneas were divided into three sections—superior, intermediate, and inferior—and each section was given a score between 0 and 3 based on the extent of the area stained^28^.

### Tear ferning test

The tear ferning test was used to detect changes in tear components, such as mucin deficiency. One microliter of tear sample was collected from the lateral canthus and dropped on a new glass slide. After the samples were dried for 10 min at room temperature (25 °C) and humidity (50%), they were observed under a light microscope (Eclipse 50i; Nikon, Tokyo, Japan). The images of the ferning pattern were graded using a five-point grading scale in increments of 0.5^29^.

### Enzyme-linked immunosorbent assay (ELISA)

For the measurement of MT levels, blood samples were taken from mice at ZT4 after 30 days of jet lag induction. The samples were placed at 4 °C overnight and then centrifuged to obtain the supernatant to obtain the serum. The concentrations of serum MT were quantified using an ELISA kit (Ameko, Shanghai, China) according to the manufacturer’s instructions. The absorbance at 450 nm was recorded by a microplate reader (BioTek, VT, USA).

### Periodic acid-Schiff (PAS) staining

After the mice were euthanized, the eyeballs were fixed with 4% paraformaldehyde. The samples were then embedded in paraffin and cut into sections along the direction of the optic nerve. A PAS staining kit (G1008-20ML; Servicebio) was used to stain the sections according to the manufacturer’s instructions. Representative images of the conjunctiva were taken by a light microscope (Eclipse 50i; Nikon). PAS-stained cells in the inferior conjunctiva were quantified.

### Immunofluorescence staining

For corneal immunofluorescence staining, mouse eyeball paraffin sections were deparaffinized, rehydrated, and subjected to antigen retrieval. Afterward, the sections were blocked with 3% bovine serum albumin (Sigma) plus 0.3% Triton X-100 (Solarbio, Beijing, China) for 1 h at room temperature. The sections were incubated with anti-MUC4 (1:50, sc-33654; Santa Cruz Biotechnology, Dallas, TX, USA) and anti-MUC1 (1:500, ab109185; Abcam, Cambridge, UK) at 4 °C overnight. After the samples were washed, they were incubated with Alexa Fluor 488-labeled anti-rabbit (1:400, 4412; Cell Signaling Technology, Danvers, MA, USA) or anti-mouse IgG antibodies (1:400, 4408; Cell Signaling Technology) for 1.5 h at room temperature and counterstained with 4’,6-diamidino-2-phenylindole (DAPI; Sigma). Images were acquired using an inverted fluorescence microscope (DMI 8; Leica, Wetzlar, Germany).

For cell immunofluorescence staining, HCE-2 cells on 24-well plates were fixed with 4% paraformaldehyde for 10 min, and the remaining steps were performed as described for corneal immunofluorescence staining. The following antibodies were used: anti-MUC4 (1:50, sc-33654; Santa Cruz Biotechnology) and anti-BMAL1 (1:200, ab3350; Abcam).

### Terminal deoxynucleotidyl transferase-mediated dUTP nick-end labeling (TUNEL) assay

A TUNEL FITC Apoptosis Detection Kit (Vazyme Biotechnology, Nanjing, China) was used to detect corneal epithelial apoptosis according to the manufacturer’s instructions. In brief, mouse eyeball sections were dewaxed, rehydrated, permeabilized with proteinase K, and then incubated with equilibration buffer for 20 min. Afterward, a TUNEL reaction mixture was used to stain the samples at 37 °C for 1 h. Finally, corneal images were obtained under an inverted fluorescence microscope (DMI 8; Leica). We manually counted the TUNEL-positive cells and used ImageJ software to determine the number of DAPI-stained cells. The percentage of TUNEL-stained cells relative to the percentage of DAPI-stained cells was calculated.

### Cell culture

The human SV40 immortalized corneal epithelial cell line (CRL-11135, human corneal epithelium [HCE-2]) was obtained from ATCC (Manassas, VA, USA) and cultured on plates in a humidified atmosphere containing 5% carbon dioxide at 37 °C. The culture medium used was Dulbecco’s modified Eagle’s medium/F12 (DMEM/F12; Gibco, Carlsbad, CA, USA) supplemented with 5 μg/mL insulin, 10 ng/mL human epidermal growth factor (Sigma), 10% fetal bovine serum, and 1% penicillin/streptomycin (Thermo Fisher Scientific; HyClone, Logan, UT, USA).

### RNA silencing

Three pairs of siRNAs targeting the human BMAL1 gene were used to silence BMAL1 expression in HCECs. The sequences of the BMAL1 siRNAs used were as follows: #1, sense, 5′-GGUUAUCCAUAUUCUGAUATT-3′, antisense, 5′-UAUCAGAAUAUGGAUAACCTT-3′; #2, sense, 5′-GCUGGAUGAAGACAACGAATT-3′, antisense, 5′-UUCGUUGUCUUCAUCCAGCTT-3′; and #3, sense, 5′-GGACCAAGGAAGUAGAAUATT-3′, antisense, 5′-UAUUCUACUUCCUUGGUCCTT-3′. A nontargeting scramble siRNA was used as a negative control treatment (Tsingke Biotechnology, Beijing, China). In brief, siRNA and Lipofectamine RNAiMAX (Invitrogen, Carlsbad, CA, USA) were added to Opti-MEM (Invitrogen). Solutions of siRNA and Lipofectamine RNAiMAX were then mixed and incubated at room temperature. Equal volumes of the mixture were added to the culture plates and cultured for 24 h.

### BMAL1 overexpression

The BMAL1-specific overexpression vector and empty vector were constructed by Tsingke Biotechnology (Beijing, China). The BMAL1 gene was cloned from PDS279_pL-CMV-GFP-ccdB-puro using Nhe I and Asc I restriction sites. Recombinant lentivirus plasmids were transfected into 293 T cells to produce recombinant lentivirus. The lentiviral vector was then transfected into HCECs as directed by the manufacturer. In brief, HCECs were seeded in cell culture plates, cultured to 40–60% confluence, and transfected with lentivirus and polybrene (Tsingke Biotechnology) for 36 h.

### RNA sequencing (RNA-seq) analysis

Total RNA was extracted from mouse cornea samples using an RNeasy Plus Mini Kit (Qiagen, Germany) following the manufacturer’s instructions, and RNA integrity was assessed with an Agilent 4200 TapeStation (Agilent Technologies, Santa Clara, CA, USA). Qualified total RNA was further purified with an RNAClean XP Kit (Beckman Coulter, CA, USA) and an RNase-Free DNase Set (Qiagen). A VAHTS Universal V6 RNA-seq Library Prep kit for Illumina (Vazyme Biotechnology) was used to construct the RNA-seq libraries. A NovaSeq 6000 platform (Illumina, San Diego, CA, USA) was used for sequencing, and 150 bp paired-end sequences were used for sequencing. The raw reads were filtered by Seqtk before they were mapped to the genome using HISAT2 (version 2.0.4). The gene fragments were counted using StringTie (v1.3.3b) followed by normalization of the trimmed mean of the M values. Significantly differentially expressed genes with a Q value < 0.05 and an absolute value of log_2_(fold change) >1 were identified using edgeR software. Batch effects were removed through the “limma” package.

### Quantitative real-time PCR (qRT‒PCR)

According to the manufacturer’s instructions, an RNeasy Plus Mini Kit Total RNA (Qiagen) was used to extract total RNA from mouse corneal tissue, and a PureLink RNA Isolation Kit (Invitrogen) was used to extract total RNA from HCECs. The RNA concentration was determined by an ND-1000 spectrophotometer (Thermo Fisher Scientific). A HiScript II 1st Strand cDNA Synthesis Kit (Vazyme Biotechnology) was used to reverse transcribe the RNA to cDNA. qRT‒PCR was performed using SYBR Green reagents (Vazyme Biotechnology). The results were analyzed by the comparative threshold cycle method, and GAPDH was used as an endogenous reference gene. The sequences of the primers used are shown in Supplementary Table 1.

### Western blot analysis

Protein samples were extracted from corneas and HCECs. Briefly, corneas and cells were lysed with RIPA lysis buffer (Fude Biological Technology, Hangzhou, China) containing 1% proteinase inhibitor (Thermo Fisher Scientific), and the supernatant was obtained by centrifugation. Afterward, the protein concentration of the samples was determined using a bicinchoninic acid protein assay kit (Thermo Fisher Scientific). Equal amounts of protein were loaded on 12% sodium dodecyl sulfate‒polyacrylamide gels and, after electrophoresis, were transferred to 0.22 μm polyvinylidene fluoride membranes (Millipore, Bedford, MA, USA). Then, the membranes were blocked with 5% fat-free milk for 1.5 h. The membranes were incubated with the following primary antibodies at 4 °C overnight: anti-MUC4 (1:100; Santa Cruz), anti-MUC1 (1:500, Abcam), anti-BMAL1 (1:200, Abcam), anti-β-actin (1:1000, Proteintech, Rosemont, IL, USA), and anti-GAPDH (1:1000, Proteintech). After the membranes were washed, they were incubated with HRP-conjugated anti-rabbit IgG (1:5000, 7074; Cell Signaling Technology) or anti-mouse IgG (1:5000, 7076; Cell Signaling Technology) for 2 h. The protein bands were visualized with an enhanced chemiluminescence kit (Beyotime Biotechnology, Shanghai, China). The band grayscale values were analyzed by ImageJ software (Version 1.53a; the National Institutes of Health).

### Statistical analysis

Prism 8.0 software (GraphPad Software, Inc., San Diego, CA, USA) was used for the statistical analyses. All the data are presented as the means ± SDs. Student’s *t* test was used for statistical comparisons between two individual groups. One- or two-way ANOVA followed by the Bonferroni post hoc correction was used for comparisons among three or more groups. *P* < 0.05 was considered to indicate statistical significance.

## Results

### Chronic jet lag leads to DED

**Fig. 1:**
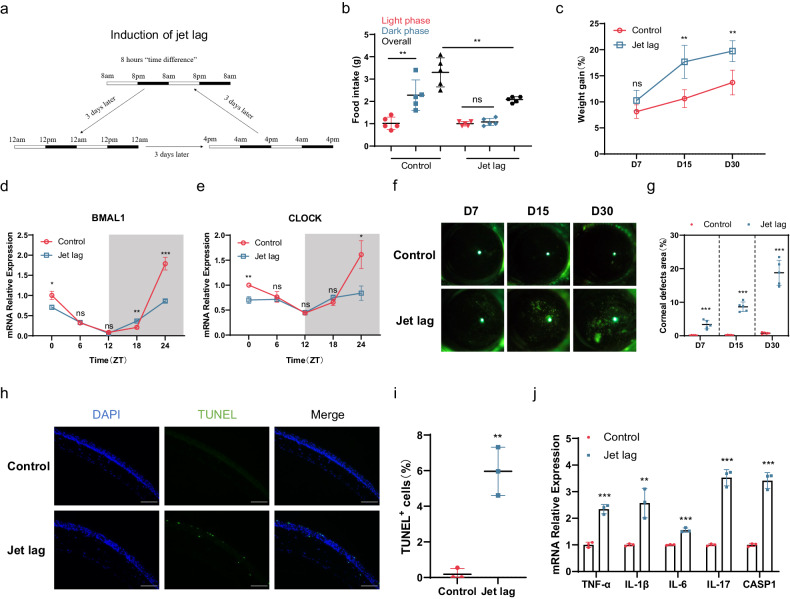
Systemic and ocular surface changes after jet lag in mice. **a** Schematic diagram of the induction of chronic jet lag. The mice were subjected to an 8 h forward time shift every 3 days. The control mice were subjected to a constant light‒dark cycle. **b** Food intake of the control mice and the mice with jet lag during the light and dark phases and overall on Day 30 (*n* = 5 mice/group). **c** Weight gain percentages of the control mice and the mice with jet lag at 7, 15, and 30 days after time shifts (*n* = 5 mice/group). **d**, **e** BMAL1 and CLOCK mRNA expression levels in corneas after 30 days of time shifts (*n* = 3 mice at each time point/group). **f**, **g** Representative examples of fluorescein sodium staining and the percentages of corneal defect area in the control mice and the mice with jet lag after 7, 15, and 30 days of time shifts (*n* = 5 mice/group). **h**, **i** Representative and quantitative results for TUNEL-positive cells in corneas after 30 days of time shifts (*n* = 3 mice/group). Scale bar = 100 μm. **j** TNF-α, IL-1β, IL-6, IL-17, and CASP1 mRNA expression levels in the cornea after 30 days of time shifts (*n* = 3 mice/group). The data are expressed as the means ± SDs. *P < 0.05, **P < 0.01, ***P < 0.001, ns: not significant. ZT = zeitgeber time.

To explore whether circadian disruption contributes to DED, we established a chronic jet lag model in which mice were exposed to 8 h time advance every 3 days to mimic circadian disruption (Fig. 1a). The establishment of a circadian disruption model was confirmed by the irregular patterns of diurnal food intake rhythms and weight gain rates. Chronic jet lag resulted in the disappearance of the dominant feeding pattern at night in the mice and a decrease in the overall daily amount of food intake (Fig. 1b). Nonetheless, compared with the control mice, the mice with jet lag exhibited greater weight gain (Fig. 1c). These phenomena were consistent with the results of a previously published study^30^. We also confirmed the successful induction of circadian disruption by detecting peripheral clock transcript oscillations, such as abnormal oscillatory expression of BMAL1 and CLOCK, in the cornea (Fig. 1d, e).

Furthermore, to investigate the effect of jet lag on DED, we performed corneal fluorescein staining and determined the tear secretion volume. The corneal defect areas gradually increased from Day 7 to Day 30 in the jet lag group (Fig. 1f, g). Interestingly, we found that there was no significant difference in tear secretion volume and only a slight shift in tear secretion rhythm between the control and jet lag groups (Supplementary Fig. 1a, b). Therefore, hematoxylin–eosin staining was performed to observe lacrimal gland structure. Unfortunately, neither the acini of the lacrimal gland nor the lacrimal duct showed significant abnormalities (Supplementary Fig. 1c). Hence, we focused on ocular surface defects. The density of apoptotic cells in the cornea was greater in the jet lag group than in the control group (Fig. 1h, i). The expression levels of inflammatory cytokines, including TNF-α, IL-1β, IL-6, IL-17, and CASP1, in the cornea were also significantly greater in the jet lag group than in the control group (Fig. 1j). These results strongly suggest that circadian disruption induces DED, including an increase in corneal epithelial cell apoptosis and the activation of inflammation.

### Chronic jet lag decreases the expression of the transmembrane mucin MUC4 in the cornea

**Fig. 2:**
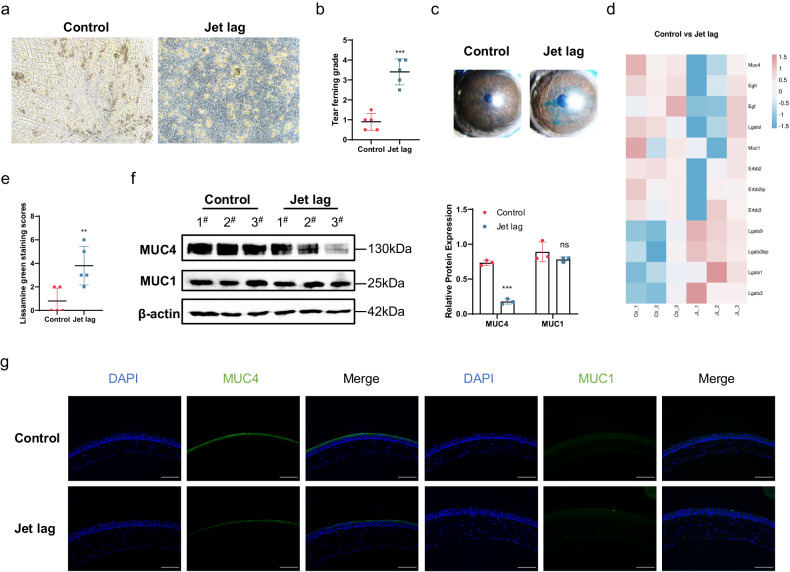
Changes in ocular surface mucins after chronic jet lag in mice. Mice with jet lag were subjected to time shifts for 30 days. **a** Representative images of tear ferning patterns in control mice and jet lag model mice (*n* = 5 mice/group). **b** Tear ferning grades (*n* = 5 mice/group). **c** Representative images of corneal lissamine green-stained samples from control mice and mice with jet lag (*n* = 5 mice/group). **d** Heatmap showing transcriptional changes in ocular surface mucins and related genes in the corneas of control mice and mice with jet lag (*n* = 3 mice/group). **e** Cumulative scores of corneal lissamine green staining (*n* = 5 mice/group). **f** Immunoblots showing the expression of MUC4 and MUC1 in the cornea. The relative protein expression levels were normalized to those of β-actin (*n* = 3 mice/group). **g** Representative immunofluorescence staining of MUC4 and MUC1 in the cornea (*n* = 3 mice/group). Scale bar = 100 μm. The data are expressed as the means ± SDs. **P < 0.01, ***P < 0.001, ns: not significant.

We further assessed the effect of jet lag on the ocular surface through a tear ferning test and lissamine green staining. The images showed that tear ferning was abnormal and that the ferning pattern grade increased in the jet lag group (Fig. 2a, b). Similarly, the lissamine green staining scores were elevated after jet lag (Fig. 2c, e). These findings suggested that abnormal tears, corneal epithelial defects, and mucin deficiency may be present in the jet lag group. Given the crucial role of the gel-forming mucin MUC5AC in ocular surface mucins, the expression of MUC5AC in the conjunctiva was measured. Interestingly, the mRNA expression of MUC5AC in the conjunctiva increased rather than decreased (Supplementary Fig. 2a). To verify our hypothesis, we used PAS staining to determine the number of goblet cells that produced MUC5AC in the conjunctiva. However, jet lag had no effect on conjunctival goblet cells (Supplementary Fig. 2b, c).

Excluding the role of the conjunctiva in mucin deficiency, we focused on the cornea. Mucins expressed in the cornea are transmembrane proteins, and one of the most important functions of transmembrane mucin is aqueous tear film anchorage^31^. Thus, bulk RNA-seq was performed on the corneas to compare the jet lag group with the control group. A heatmap indicated that the expression levels of mucin genes and related genes were reduced in the jet lag group (Fig. 2d). To verify the changes in the expression of mucins, we assessed MUC1 and MUC4 at the protein level. Interestingly, the protein expression of MUC4 was significantly decreased in the jet lag group, while that of MUC1 did not change (Fig. 2f, g). We also noted that MUC4 was only expressed by stratified squamous epithelia along the apical membrane of the apical surface of the cornea, which may contribute to the function of MUC4 (Fig. 2g). These results suggest that circadian disruption leads to deficiencies of corneal transmembrane mucins, specifically MUC4.

### Supplemental MUC4 reverses jet lag-induced dry eye

**Fig. 3:**
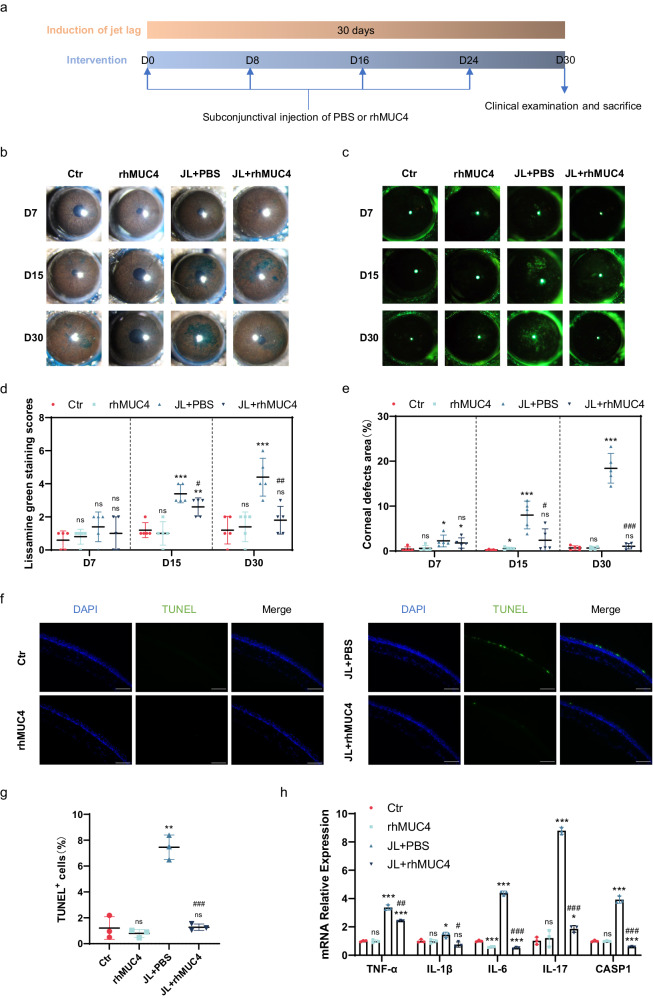
Effect of rhMUC4 on dry eye in mice subjected to jet lag. **a** Schematic showing the workflow of rhMUC4 treatment in control mice and mice with jet lag. **b** Representative images of corneal lissamine staining at 7, 15, and 30 days after PBS or rhMUC4 treatment in control mice and mice with jet lag (*n* = 5 mice/group). **c** Representative samples of corneal fluorescein sodium staining from control mice and mice with jet lag after treatment with PBS or rhMUC4 on Days 7, 15, and 30 (*n* = 5 mice/group). **d** Cumulative scores of corneal lissamine green staining after PBS or rhMUC4 treatment (*n* = 5 mice/group). **e** Percentages of corneal defect area in the control mice and the mice with jet lag treated with PBS or rhMUC4 after 7, 15, and 30 days of time shifts (*n* = 5 mice/group). **f** Representative images of TUNEL staining of corneas from control mice and mice with jet lag after PBS or rhMUC4 treatment (*n* = 3 mice/group). Scale bar = 100 μm. **g** Percentages of corneal cells stained with TUNEL after treatment with PBS or rhMUC4 in the control mice and the mice with jet lag (*n* = 3 mice/group). **h** TNF-α, IL-1β, IL-6, IL-17, and CASP1 mRNA expression levels in the cornea after 30 days of time shifts with PBS or rhMUC4 treatment (*n* = 3 mice/group). The data are expressed as the means ± SDs. *P < 0.05, **P < 0.01, ***P < 0.001 versus the untreated control group. ^#^*P* < 0.05, ^##^*P* < 0.01, ^###^*P* < 0.001 versus the jet lag with PBS treatment group. ns: not significant. Ctr = control; JL = jet lag; PBS = phosphate-buffered saline; rhMUC4 = recombinant human MUC4.

Given the significant decrease in MUC4 in the cornea in the chronic jet lag model, we explored whether MUC4 is a critical molecule in jet lag-induced dry eye. Therefore, MUC4 was administered 4 times by subconjunctival injection of rhMUC4 during the induction of chronic jet lag (Fig. 3a). To investigate the effect of supplemental MUC4 on jet lag-induced dry eye, we performed lissamine green staining and corneal fluorescein staining. Lissamine green staining was significantly decreased by rhMUC4 in the mice with jet lag (Fig. 3b, d). Moreover, the corneal defect area was significantly decreased by the addition of MUC4 in the mice subjected to jet lag (Fig. 3c, e). Furthermore, jet lag-induced corneal epithelial cell apoptosis was inhibited by the addition of MUC4 (Fig. 3f, g). The increases in inflammatory factors, including TNF-α, IL-1β, IL-6, IL-17, and CASP1, induced by jet lag were inhibited by MUC4 supplementation (Fig. 3h). In summary, chronic jet lag induces dry eye by reducing the level of the transmembrane mucin MUC4 in the cornea.

### BMAL1 KO mice show MUC4 deficiency and DED

**Fig. 4:**
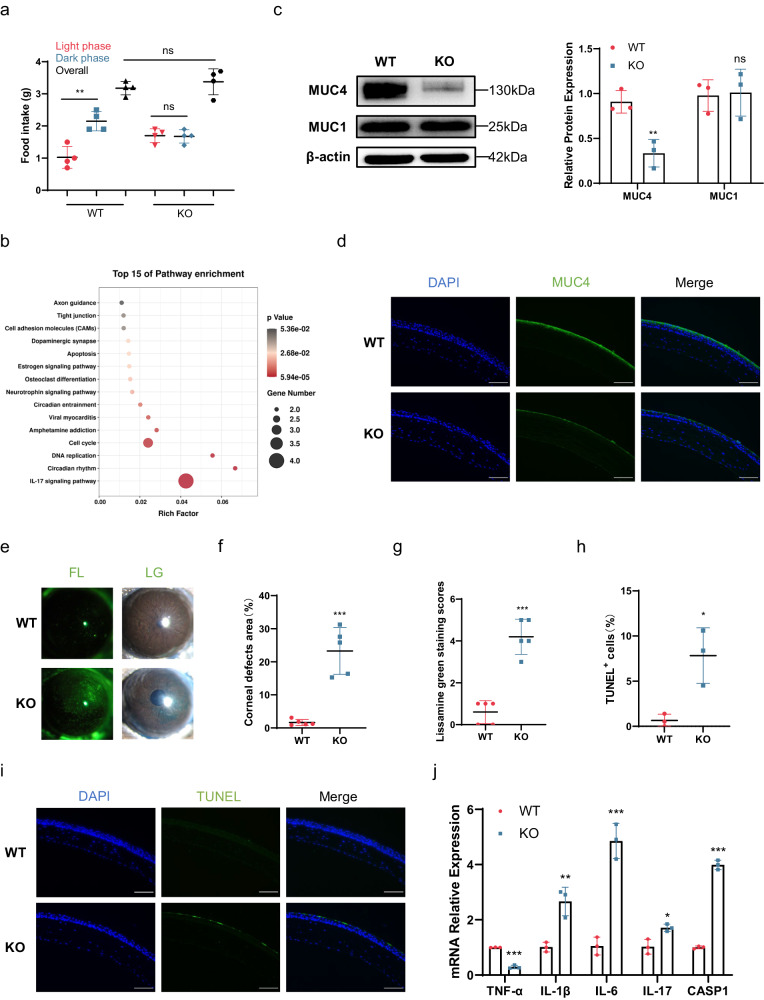
Systemic and ocular surface changes in BMAL1 KO mice. **a** Food intake of WT and BMAL1 KO mice during the light and dark phases and overall (*n* = 4 mice/group). **b** Top 15 enriched KEGG pathways in corneas from WT and KO mice (*n* = 2 mice/group). **c** Representative immunoblot images showing the protein levels of MUC4 and MUC1 in the corneas of WT and KO mice. The relative protein expression levels were normalized to those of β-actin (*n* = 3 mice/group). **d** Representative immunofluorescence staining of MUC4 in the cornea (*n* = 3 mice/group). Scale bar = 100 μm. **e** Representative samples of corneal fluorescein sodium and lissamine green staining from WT and KO mice (*n* = 5 mice/group). **f** Percentages of corneal defect area in the WT and KO mice (*n* = 5 mice/group). **g** Scores of corneal lissamine green staining of corneas from the WT and KO mice (*n* = 5 mice/group). **h** Percentages of TUNEL-positive cells in the cornea (*n* = 3 mice/group). **i** Representative results of TUNEL staining of corneas from WT and KO mice (*n* = 3 mice/group). Scale bar = 100 μm. **j** TNF-α, IL-1β, IL-6, IL-17, and CASP1 mRNA expression levels in the corneas of the WT and KO mice (*n* = 3 mice/group). The data are expressed as the means ± SDs. *P < 0.05, **P < 0.01, ***P < 0.001, ns: not significant. WT = wild type; KO = knockout; FL = fluorescein sodium; LG = lissamine green.

We next investigated the mechanism underlying the relationship between circadian disruption and MUC4 deficiency. The biological clock is synchronized to the diurnal variation in the environment by the molecular components of the circadian clock. Notably, BMAL1 is one of the core activators of the molecular circadian clock that function together to produce a 24-h rhythm of gene expression^32^. We found that the average expression of BMAL1 decreased in our jet lag model (Fig. 1d). Thus, we investigated whether molecular circadian clock homeostasis is required to prevent DED using BMAL1 KO mice, in which the circadian clock has been genetically disrupted. To demonstrate successful disruption of the circadian clock, we observed the diurnal food intake patterns in WT and BMAL1 KO mice. Similar to mice with jet lag, BMAL1 KO mice lost their dominant feeding pattern at night (Fig. 4a). To validate the peripheral clock transition, we performed bulk RNA-seq of corneas from the WT and KO groups. Kyoto Encyclopedia of Genes and Genomes (KEGG) pathway enrichment analysis of differentially expressed genes between the WT and KO groups revealed that circadian rhythm and circadian entrainment were enriched (Fig. 4b). Then, we explored whether ablation of BMAL1 induced transmembrane mucin deficiency and DED. We observed a significant reduction in MUC4, while MUC1 expression did not differ between the BMAL1 KO mice and the WT mice (Fig. 4c, d and Supplementary Fig. 3). Corneal fluorescein staining and lissamine green staining were used to assess corneal defects and mucin deficiency. As expected, corneal defects and mucin-deficient areas were elevated in the BMAL1 KO mice (Fig. 4e–g). Further analysis revealed corneal epithelial death and inflammatory activation in BMAL1 KO mice (Fig. 4h–j). Taken together, these data suggest that circadian disruption induces DED, presumably by reducing MUC4 expression through disruption of the circadian clock molecule BMAL1.

### BMAL1 directly regulates MUC4 in HCECs

**Fig. 5:**
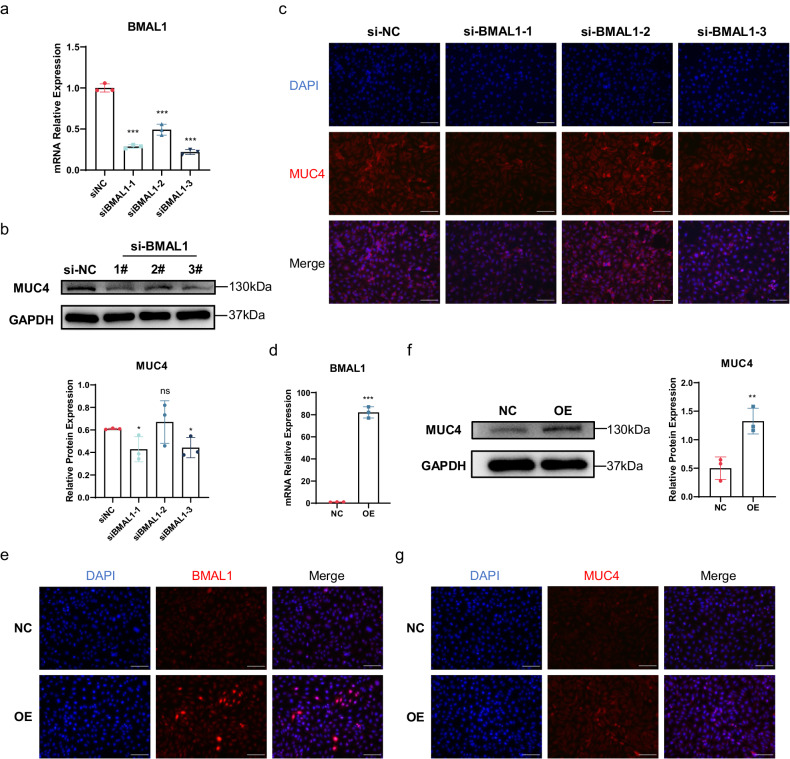
Effect of BMAL1 on MUC4 in HCECs. **a** Efficiency of BMAL1 knockdown by siRNA (*n* = 3 samples/group). **b** Representative immunoblot images showing the protein levels of MUC4 after BMAL1 knockdown in HCECs. The relative protein expression levels were normalized to those of GAPDH (*n* = 3 samples/group). **c** Representative immunofluorescence staining of MUC4 after BMAL1 knockdown in HCECs (*n* = 3 samples/group). Scale bar = 100 μm. **d** Efficiency of BMAL1 overexpression by lentivirus (*n* = 3 samples/group). **e** BMAL1 protein expression after lentivirus-mediated overexpression in HCECs (*n* = 3 samples/group). Scale bar = 100 μm. **f** Representative immunoblot images of MUC4 after BMAL1 overexpression in HCECs. The relative protein expression levels were normalized to those of GAPDH (*n* = 3 samples/group). **g** Representative immunofluorescence staining of MUC4 after BMAL1 overexpression in HCECs (*n* = 3 samples/group). Scale bar = 100 μm. The data are expressed as the means ± SDs. *P < 0.05, **P < 0.01, ***P < 0.001, ns: not significant. siRNA = small interfering RNA; NC = negative control; OE = overexpression.

Given that BMAL1 KO mice exhibit MUC4 deficiency, which promotes DED, we examined whether the clock molecule BMAL1 directly regulates the expression of MUC4. To this end, we performed confirmatory experiments in HCECs. We designed 3 pairs of siRNAs to knock down the expression of BMAL1 in HCECs, and the silencing efficiency was confirmed by qRT‒PCR (Fig. 5a). Interestingly, the expression of MUC4 significantly decreased along with the reduction in BMAL1 in HCECs (Fig. 5b, c). To further confirm the relationship between BMAL1 and MUC4, we constructed a lentiviral overexpression system for BMAL1. The BMAL1-overexpressing lentivirus significantly increased the expression of BMAL1 in HCECs (Fig. 5d, e). Surprisingly, the expression of MUC4 markedly increased after BMAL1 was overexpressed (Fig. 5f, g). Taken together, our results show that BMAL1 directly regulates the expression of MUC4 in HCECs.

### MT restores the circadian rhythm and relieves MUC4 deficiency

**Fig. 6:**
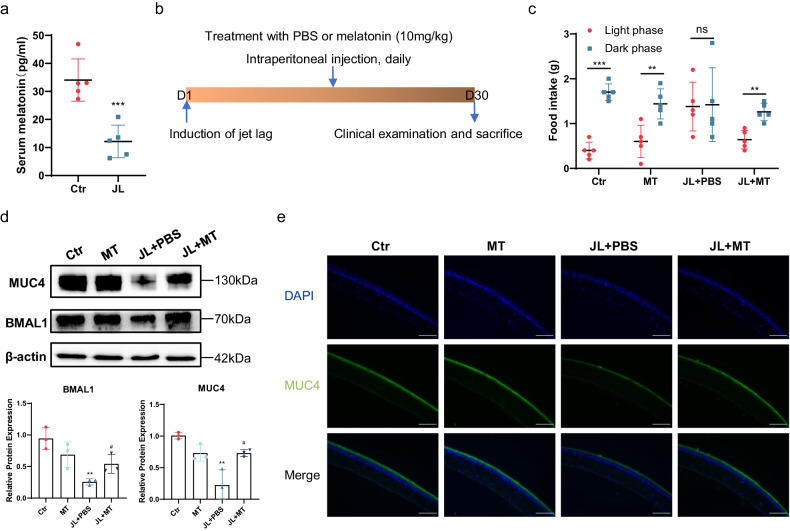
Effects of MT on circadian rhythm and MUC4 in mice with jet lag. **a** Serum MT levels in the control mice and the mice with jet lag after 30 days of time shifts (*n* = 5 mice/group). **b** Schematic showing the workflow of MT or PBS treatment in control mice and mice with jet lag. **c** Food intake of the control mice, MT-treated mice, PBS-treated mice with jet lag, and MT-treated mice with jet lag during the light and dark phases (*n* = 5 mice/group). **d** Representative immunoblots of BMAL1 and MUC4 in control mice, MT-treated mice, and jet lag-treated mice treated with PBS or MT. The relative protein expression levels were normalized to those of β-actin (*n* = 3 mice/group). **e** Representative immunofluorescence staining of MUC4 in corneas after treatment with MT or PBS (*n* = 3 mice/group). Scale bar = 100 μm. The data are expressed as the means ± SDs. **P < 0.01, ***P < 0.001 versus the untreated control group. ^#^*P* < 0.05 versus the jet lag plus PBS-treated group. ns: not significant. Ctr = control; MT = melatonin; JL = jet lag; PBS = phosphate-buffered saline.

Finally, we examined whether circadian disruption-induced DED can be treated. MT is primarily produced by the pineal gland and maintains an individual’s circadian rhythm^33^. In our previous study, we demonstrated that MT protects corneal epithelial cells from oxidative damage in a dry eye mouse model induced by scopolamine hydrobromide^34^. Thus, we first determined the serum MT level in control mice and mice with jet lag using an ELISA kit. The serum MT level was significantly lower in the jet lag group than in the control group (Fig. 6a). Therefore, we treated the mice with MT during jet lag induction (Fig. 6b). We next evaluated the therapeutic effects of MT in a jet lag model. Surprisingly, the jet lag-induced irregular pattern of diurnal food intake rhythms was reversed by MT (Fig. 6c). Furthermore, we determined the expression of BMAL1 in the cornea to assess the effect of MT on the core molecular clock. Western blotting indicated that jet lag induced a decrease in BMAL1 expression and that treatment with MT significantly elevated BMAL1 expression (Fig. 6d). Moreover, relative to PBS treatment, MT treatment markedly improved MUC4 deficiency (Fig. 6d, e). Thus, MT treatment restored the circadian rhythm and increased BMAL1 and MUC4 expression in the corneas of chronic jet lag model mice.

### MT rescues DED induced by chronic jet lag

**Fig. 7:**
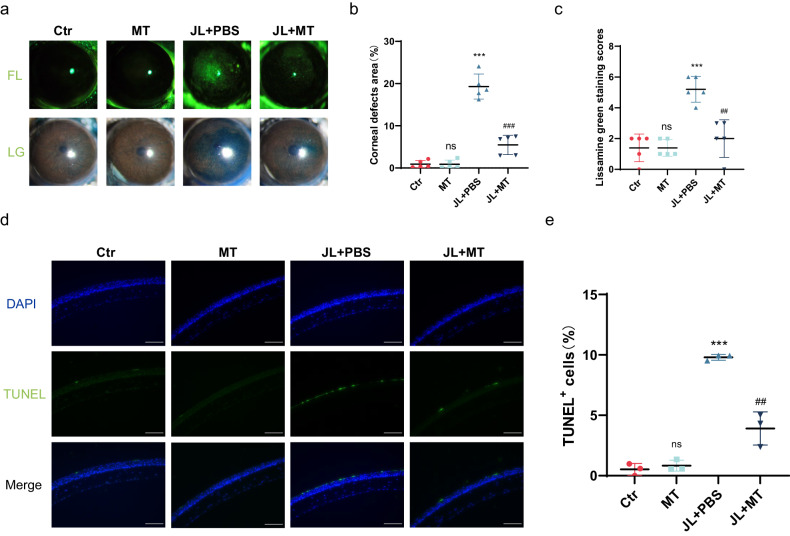
Effect of MT on dry eye in mice with jet lag. **a** Representative images of corneal fluorescein sodium and lissamine green staining in control mice, MT-treated mice, mice with jet lag treated with PBS, and mice with jet lag treated with MT (*n* = 5 mice/group). **b** Percentages of corneal defect area in the four groups (*n* = 5 mice/group). **c** Corneal lissamine green staining scores (*n* = 5 mice/group). **d** Representative TUNEL-positive cells in the corneas of control mice, MT-treated mice, mice with jet lag treated with PBS, and mice with jet lag treated with MT (*n* = 3 mice/group). Scale bar = 100 μm. **e** Quantification of TUNEL-positive cells in the corneas of the four groups. The data are expressed as the means ± SDs. ***P < 0.001 versus the untreated control group. ^##^*P* < 0.01, ^###^*P* < 0.001 versus the jet lag with PBS treatment group. ns: not significant. Ctr = control; MT = melatonin; JL = jet lag; PBS = phosphate-buffered saline.

Furthermore, we explored the effect of MT on ocular surface damage induced by circadian disruption. We first evaluated corneal epithelial defects using corneal fluorescein staining and lissamine green staining. The corneal defect area and lissamine green staining scores were significantly lower in the MT-treated mice than in the PBS-treated mice (Fig. 7a–c). Consistent with these findings, the TUNEL staining results showed that the density of apoptotic cells in the cornea was substantially lower in the MT treatment group than in the PBS treatment group (Fig. 7d, e). Taken together, these data suggest that MT has therapeutic effects on circadian disruption-induced dry eye.

## Discussion

Recently, the impact of lifestyle challenges on ocular surface diseases has attracted widespread attention^2^. However, the role of circadian disruption in DED and the underlying mechanisms involved remain unclear. The circadian rhythm has been noted to be strongly linked to human health. Circadian disruption contributes to the pathogenesis of systemic disorders such as obesity, diabetes, and cancer^21^. In addition, many ocular tissues, including the retina, lens, cornea, and choroid, exhibit active circadian rhythms to accommodate diurnal environmental variability^35^. Thus, circadian entrainment may be associated with corneal and ocular surface diseases. Moreover, a recent study confirmed that 5 days of acute jet lag impaired the circadian rhythm of the lacrimal glands, including their structure and secretion function^25^. However, the acute jet lag model does not adequately mimic the shift work and jet lag that are common in daily life. Therefore, in this study, we constructed a chronic jet lag mouse model by continuously advancing light exposure by 8 h every 3 days for 30 days. We found that chronic jet lag, unlike acute jet lag, mainly induced MUC4 deficiency, corneal epithelial cell apoptosis and inflammatory activation rather than lacrimal gland damage. The short-term changes in jet lag may be reversible, while long-term jet lag causes irreversible damage. This molecular distinction between acute and chronic jet lag is interesting and needs further exploration in the future.

Further analysis of the circadian disruption-induced dry eye model suggested that MUC4 deficiency was essential for pathogenesis. Several clinical studies have shown that alterations in mucins on the ocular surface promote DED^36,37^. In a recent study, MUC5AC expression was negatively correlated with the ocular surface disease index (OSDI)^38^. However, the role of transmembrane mucins in DED remains unclear. Some studies have suggested that MUC1 and MUC4 mRNA expression levels are significantly lower in patients with severe dry eye than in healthy subjects^39^. Other studies have shown no significant change in MUC1 and MUC4 expression between patients with dry eye and controls^36^. In our study, we observed significant reductions in both the mRNA and protein levels of MUC4 in the circadian disruption model. When mice with jet lag were treated with rhMUC4, the number of corneal apoptotic cells and the levels of corneal inflammatory cytokines were significantly reduced. Moreover, in a comparison between the control and JL+rhMUC4 groups, we found no significant difference in either the corneal defect area or apoptosis. These results indicated that MUC4 strongly regulates cell death in jet lag-induced dry eye. Similarly, some cancer studies have revealed a relationship between MUC4 and cell death^40,41^. However, we found that some inflammatory factors, such as TNF-α and IL-17, were still more highly expressed in the JL+rhMUC4 group than in the control group. Therefore, we can only demonstrate that MUC4 partially regulates chronic jet lag-induced dry eye. Although preliminary, these results provide important evidence that mucin deficiency induces corneal epithelial cell death in circadian disruption-induced DED. Furthermore, MUC4 is involved in signal transduction. MUC4 has extracellular EGF-like domains that bind to the EGF receptors Erb-B2 receptor tyrosine kinase 2 (ERBB2) and Erb-B2 receptor tyrosine kinase 3 (ERBB3). ERBB2 and ERBB3 function to induce proliferation and apoptosis in epithelial cells^31^. In the present study, we focused only on the role of MUC4 as a transmembrane mucin. However, the effect of MUC4 signal transduction on the ocular surface is still unclear. In addition, the composition of corneal transmembrane mucins varies between humans and mice. MUC1, MUC4, and MUC16 are expressed in human corneas, but MUC16 expression is absent in mouse corneas and is replaced by that of MUC4^31^. Thus, the MUC4 deficiency caused by circadian disruption that we observed in mice is not fully representative of the situation in humans, and further studies in humans are needed to validate our findings.

To further validate the regulatory relationship between the molecular clock and MUC4 deficiency, we used BMAL KO mice, which lose their circadian rhythm at birth. BMAL1 KO is the only single KO that leads to the elimination of clock function in both suprachiasmatic nuclei and peripheral tissues. BMAL1 KO mice lack all molecular and behavioral circadian phenotypes and present reduced activity and body weight and a shortened lifespan^20^. Here, BMAL1 KO mice exhibited MUC4 deficiency, corneal damage and inflammatory activation. However, it is difficult to determine whether MUC4 deficiency is due to BMAL1 ablation or systemic circadian rhythm loss. Furthermore, previous studies have shown that rescuing the expression of BMAL1 solely in muscle tissue normalized activity levels, body weight, and longevity in BMAL1 KO mice, though these animals remained behaviorally arrhythmic^42^. These surprising results indicate that local control of the peripheral clock is sufficient to drive tissue-specific physiology. The function of the peripheral clock in the cornea and other ocular surface tissues remains to be further explored.

In addition to establishing a mouse model of circadian disruption-induced dry eye and exploring the underlying mechanisms, we also attempted to identify a possible treatment. MT is an indoleamine-containing hormone that is primarily secreted by the pineal gland to maintain circadian rhythm^43^. Interestingly, MT receptors have been identified in many ocular tissues, including the neural retina, retinal pigment epithelium, ciliary body, cornea, sclera, and lens^35^. Therefore, we applied MT to circadian disruption and found that the circadian rhythm of food intake was restored. Furthermore, the expression levels of BMAL1 and MUC4 were significantly elevated in the cornea, and the symptoms of DED were alleviated. MT has powerful antioxidant and anti-inflammatory properties^44^. As a result, many studies have demonstrated that MT can treat a variety of diseases, such as heart failure^45^, cancer^46^, and diabetic retinopathy^47^. Indeed, in our previous research, MT protected corneal epithelial cells from oxidative damage by triggering heme oxygenase-1 (HO-1) expression in another dry eye mouse model induced by scopolamine hydrobromide^34^. Thus, we identified two distinct pathways involved in the treatment of DED with MT.

Collectively, our data demonstrate that circadian disruption induces corneal epithelial apoptosis and ocular surface inflammation by inhibiting the BMAL1–MUC4 axis, and MT restores ocular surface homeostasis by alleviating the inhibition of the BMAL1–MUC4 axis. These findings identified a novel dry eye mouse model induced by circadian disruption, elucidated the underlying mechanism and identified a potential clinical treatment.

### Supplementary information

Supplementary Information
