# Supplementary material for: Circadian disruption reduces MUC4 expression via the clock molecule BMAL1 during dry eye development
Source: Exp Mol Med. 2024 Jul 2;56(7):1655–66. doi: 10.1038/s12276-024-01269-0 (PMC11297157; doi:10.1038/s12276-024-01269-0)
Supplement: Supplementary file 1 — Supplementary Information [file 12276_2024_1269_MOESM1_ESM.pdf]

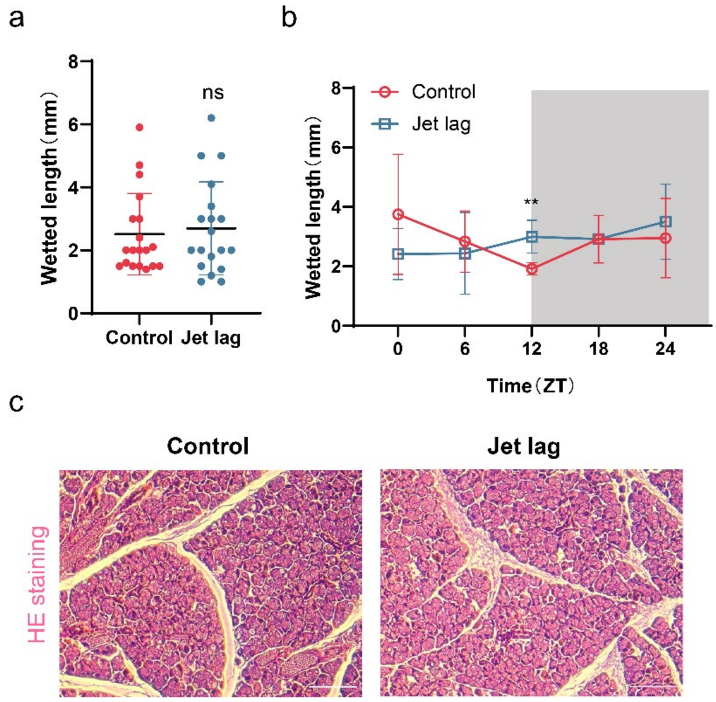

Supplementary Fig. 1 Changes in the function and structure of the lacrimal gland after time shifts in mice. **a** Phenol thread tear production test in the control mice and the mice with jet lag (n = 19 mice/group). **b** Phenol thread tear production test in a 24 hours cycle between control and jet lag groups (n = 6 mice/group). **c** Representative HE staining images of lacrimal gland in the control mice and the mice with jet lag (n = 3 mice/group). Scale bar = 100 μm. The data are expressed as the means ± SDs. **p < 0.01, ns: not significant.

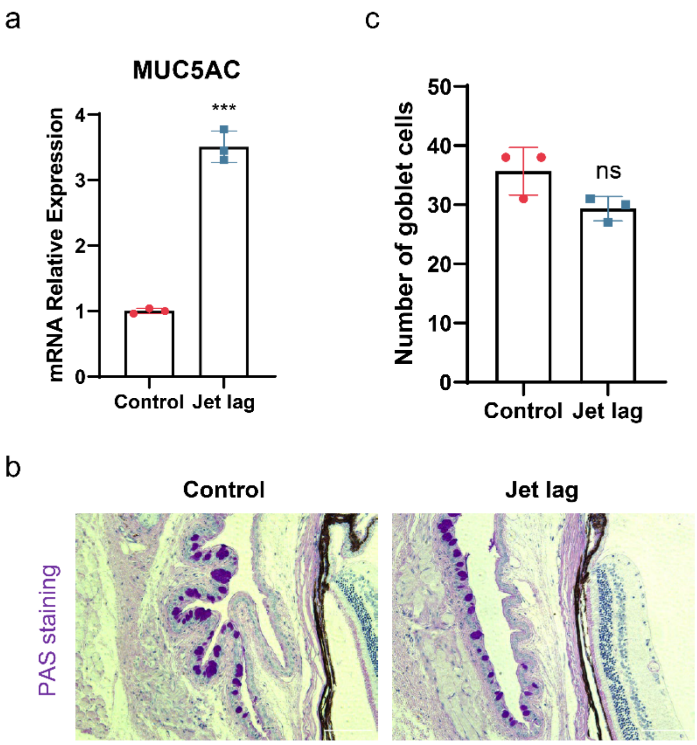

Supplementary Fig. 2 Conjunctival function changes in mice with jet lag. a Conjunctiva MUC5AC mRNA levels in the control mice and the mice with jet lag (n = 3 mice/group). **b** Representative PAS staining results of conjunctiva in the control mice and the mice with jet lag (n = 3 mice/group). Scale bar = 100 μm. **c** Number of goblet cells in conjunctiva in the control mice and the mice with jet lag (n = 3 mice/group). The data are expressed as the means ± SDs. ***p < 0.001, ns: not significant.

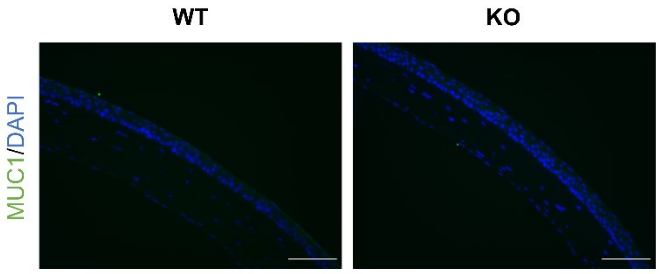

Supplementary Fig. 3 Representative immunofluorescence staining of MUC1 in cornea in the WT and BMAL1 KO mice (n = 3 mice/group).

Supplementary Table 1. Primer sequences for qRT-PCR

Primer	Forward (5’-3’)	Reverse (5’-3)
mBMAL1	ACAGTCAGATTGAAAAGAGGCG	GCCATCCTTAGCACGGTGAG
mCLCOK	ATGGTGTTTACCGTAAGCTGTAG	CTCGCGTTACCAGGAAGCAT
mTNF-α	CCTGTAGCCCACGTCGTAG	GGGAGTAGACAAGGTACAACCC
mIL-1β	GCAACTGTTCCTGAACTCAACT	ATCTTTTGGGGTCCGTCAACT
mIL-6	TAGTCCTTCCTACCCCAATTTCC	TTGGTCCTTAGCCACTCCTTC
mIL-17	TTTAACTCCCTTGGCGCAAAA	CTTTCCCTCCGCATTGACAC
mCASP-1	GGCACATTTCCAGGACTGACTG	GCAAGACGTGTACGAGTGGTT
mMUC5AC	CAGGACTCTCTGAAATCGTACCA	GAAGGCTCGTACCACAGGG
mGAPDH	CATCACTGCCACCCAGAAGACT	ATGCCAGTGAGCTTCCCGTTCA
hBMAL1	AAGGGAAGCTCACAGTCAGAT	GGACATTGCGTTGCATGTTGG
hGAPDH	GTCTCCTCTGACTTCAACAGC	ACCACCCTGTTGCTGTAGCCA
